# Current progress on innate immune evasion mediated by N^pro^ protein of pestiviruses

**DOI:** 10.3389/fimmu.2023.1136051

**Published:** 2023-04-05

**Authors:** Shubo Wen, Xintong Li, Xiangyu Lv, Kai Liu, Jingqiang Ren, Jingbo Zhai, Yang Song

**Affiliations:** ^1^ Preventive Veterinary Laboratory, College of Animal Science and Technology, Inner Mongolia Minzu University, Tongliao, China; ^2^ Key Laboratory of Zoonose Prevention and Control, Universities of Inner Mongolia Autonomous Region, Tongliao, China; ^3^ Wenzhou Key Laboratory for Virology and Immunology, Institute of Virology, Wenzhou University, Zhejiang, Wenzhou, China; ^4^ State Key Laboratory of Membrane Biology, Institute of Zoology, Chinese Academy of Sciences, Beijing, China; ^5^ Beef Cattle Disease Control and Engineering Technology Research Center, Inner Mongolia Autonomous Region, Tongliao, China

**Keywords:** pestivirus, interferon (IFN), immune evasion, viral proteins, innate immunity

## Abstract

Interferon (IFN), the most effective antiviral cytokine, is involved in innate and adaptive immune responses and is essential to the host defense against virus invasion. Once the host was infected by pathogens, the pathogen-associated molecular patterns (PAMPs) were recognized by the host pattern recognition receptors (PRRs), which activates interferon regulatory transcription factors (IRFs) and nuclear factor-kappa B (NF-κB) signal transduction pathway to induce IFN expression. Pathogens have acquired many strategies to escape the IFN-mediated antiviral immune response. Pestiviruses cause massive economic losses in the livestock industry worldwide every year. The immune escape strategies acquired by pestiviruses during evolution are among the major difficulties in its control. Previous experiments indicated that Erns, as an envelope glycoprotein unique to pestiviruses with RNase activity, could cleave viral ss- and dsRNAs, therefore inhibiting the host IFN production induced by viral ss- and dsRNAs. In contrast, Npro, the other envelope glycoprotein unique to pestiviruses, mainly stimulates the degradation of transcription factor IRF-3 to confront the IFN response. This review mainly summarized the current progress on mechanisms mediated by Npro of pestiviruses to antagonize IFN production.

## Introduction

The genus Pestivirus, belonging to the family Flaviviridae, comprises pathogens responsible for massive economic losses in livestocks, especially pigs and ruminant species (1–3) and often cause clinical manifestations ranging from mild to severe (4–7). Among pestiviruses, classical swine fever virus (CSFV) and bovine viral diarrhea virus 1 and 2 (BVDV-1 and BVDV-2) are the most impactful ones. Notably, the majority of pestiviruses are non-cytopathogenic (NCP), while both two biological types: NCP and cytopathogenic (CP) viruses have been reported in CSFV and BVDV strains isolated in clinical samples (4). Several other viruses related to pestivirus have been also described in some studies. These viruses isolated from domestic animals (8–17) and wild species (18–21) have great differences in genetics. Recently, the following eleven viruses: BVDV-1, BVDV-2, CSFV, BDV, pronghorn pestivirus, Bungowannah virus, giraffe pestivirus, HoBi-like pestivirus, Aydin-like pestivirus, rat pestivirus, and atypical porcine pestivirus have been appointed to Pestivirus A-K, respectively (3, 15).

Pestiviruses could transmit from one species of ruminants to another frequently. For example, ruminant pestiviruses often infect pigs (22). However, no evidence is available to suggest the replication of CSFV in ruminants. After infection, pestiviruses are excreted through various body secretions and usually transmitted by direct contact with infected animals or indirect contact with infectious secretions, contaminated food, or needles (23). Animals with pestiviruses infection (especially those with persistent infection) excrete lots of viruses from their body secretions for life.

Pestiviruses are single-stranded, positive-sense RNA viruses with an envelope and a genomic size of about 12.3 kb (24). The genomic RNA is translated into a single polyprotein, which is processed subsequently into four structural proteins (SPs): a basic core protein C and three envelope (E) glycoproteins E^rns^, E1, and E2, as well as eight non-structural proteins. Proteins unique to the Pestivirus genus are the non-structural protease N^pro^ and E^rns^ envelope glycoprotein which has RNase activity. Both proteins are associated with the suppression of the host’s innate antiviral immune response (23). This review compiles current progresses on the roles and functions of Pestivirus N^pro^ in the evasion of type I interferon response.

## N^pro^ of pestiviruses has antagonistic activity against type-I IFN production

N^pro^ is the first protein encoded by pestivirus, with a molecular weight of 23 KDa, and is unique to the pestivirus genus. N^pro^ protein is a hydrophilic peripheral membrane protein without signal peptide, and the secondary structure mainly contains β-sheet and random curling. Moreover, it has autoprotease activity and can be cleaved in an autocatalytic manner from nascent polyproteins being translated into mature viral proteins. N^pro^ is not necessary for the replication of pestiviruses but plays an important role in the evasion of the antiviral immune response of host cells. It has been shown that Cys69 and His130 are the catalytic residues of protease cleavage and catalyze the cleavage of peptide bonds between Tyr164 and Vail65. To study the biological activity of N^pro^, Tratschin et al. prepared a CSFV virus strain vA187-Ubi, the N^pro^ protein gene sequence of which was replaced by the mouse ubiquitin protein gene. It has been shown that vA187-Ubi had similar growth characteristics to the parent vA187-1 virus, both of which showed obvious cytopathological effects. *In vivo* assay results showed a complete loss of virulence of vA187-Ubi, indicating the N^pro^ protein is unnecessary for the virus replication but is essential for its virulence (25). In addition, the N^pro^ protein of pestiviruses could block apoptosis and IFN-α/β production induced by double-stranded RNA. NCP-type BVDV-1 infection was found to protect bovine nose osteocytes from poly (I: C) -induced apoptosis. Further studies showed that NCP-type BVDV inhibited the transcription and secretion of type I interferon-induced poly (I: C) (26). In addition, compared with the parent strain, ΔN^pro^ BVDV can effectively induce IFN-β production, indicating that N^pro^ could inhibit the production of type I interferon (27). The N^pro^ protein of BVDV-2 could also significantly down-regulate oligo adenylate synthetase (OAS), ubiquitin-like protein 15 (ISG15), Myxoviral-resistant protein 1 (Mx1), and type I IFN transcription levels (28).

Studies on CSFV have shown similar results. Ruggli et al. reported that after infection with CSFV, the resistance to poly (I: C) -induced apoptosis by porcine renal cell line SK-6 increased nearly 100 times. ΔN^pro^ CSFV was found to have a similar growth profile to wild-type virus, but with no protection for SK-6 cells against apoptosis induced by poly (I: C) (29). Therefore, it was suggested that N^pro^ could counteract dsRNA-induced apoptosis and IFN-α/β production independently of other CSFV proteins. After treated with poly (I: C), drastically more SK6-EGFP-N^pro^ cells and CSFV-infected SK-6 cells survived compared with the parental SK-6 cells. Luciferase reporter gene experiments showed that N^pro^ also inhibits the expression of luciferase derived by IFN-α/β promoter in human cells, meanwhile, it can also inhibit the production of Newcastle disease virus-induced IFN-α/β (30). Moreover, in dendritic cells (DC), ΔN^pro^ CSFV can promote the expression of IFNα/β, and also up-regulate the expression of CD80/86 and MHC II to promote the maturation of DCs (31).

Bungowannah virus is genetically the most divergent pestivirus with all of the genomic and structural elements of classical pestiviruses. Compared with other pestivirus, they also have many differences in antigenic cross-reaction. To test the influence of N^pro^ of Bungowannah virus on the type I interferon signaling pathway, a chimeric BVDV/Bungowannah virus (vCP7_N^pro^-Bungo) was rescued by Richter et al. (32). In the virus, the N^pro^ gene of Bungowannah virus replaced that of CP7— a cytopathic BVDV strain. After infected with CP7, Bungowannah virus, and virus vCP7_Npro-Bungo, similar IFN suppression was observed in cells. However, the Npro-deleted mutant had an impaired replication and induced increased type-I IFN response in bovine cells (32). Collectively, these studies indicated that the N^pro^ of pestiviruses had antagonistic protease activity of IFN-α/β production.

Furthermore, it has been shown that the replacement of amino acids Glu22 and His49 of pestiviruses could abolish the ability of N^pro^ to inhibit IFN production, while the replacement of Cys69 had no such effect. There was no antagonistic IFN-α/β activity in the conserved N^pro^ region (L8P) mutant near the N-terminal of the two BVDV biotypes, demonstrating the integrality of the N^pro^ N-terminal structure is essential in the catalytic activity of IFN-α/β inhibition (33).

## N^pro^ induces proteasome degradation of IRF3

### Overview of IRF3

The IRF family has been reported to have 10 members, namely IRF1-IRF9 and virus IRF (v-IRF). IRF3, a principal transcription factor, is significant in the antiviral immune response (34). IRF3 is highly homologous to IRF7. Both of them regulate the type I IFN synthesis, but play different roles in the innate immune response. IRF3 is critical for early induction of IFN expression in most cells post-viral infection; IRF7, which induces both IFNα and IFNβ expression, has functions in the antiviral activity of IFN in a later stage. In contrast, IRF3 can induce IFNβ gene expression, but not other IFNα expression except IFNα4 (35). Upon viral infection, a series of cellular pathways are activated subsequently to promote the translocation of phosphorylated IRF3 or IRF7 into the nucleus and initiate the transcription of type I interferon genes by attaching to IFN-α/β promoters (36).

### N^pro^ mediates ubiquitination and proteasomal degradation of IRF3

**Figure 1:**
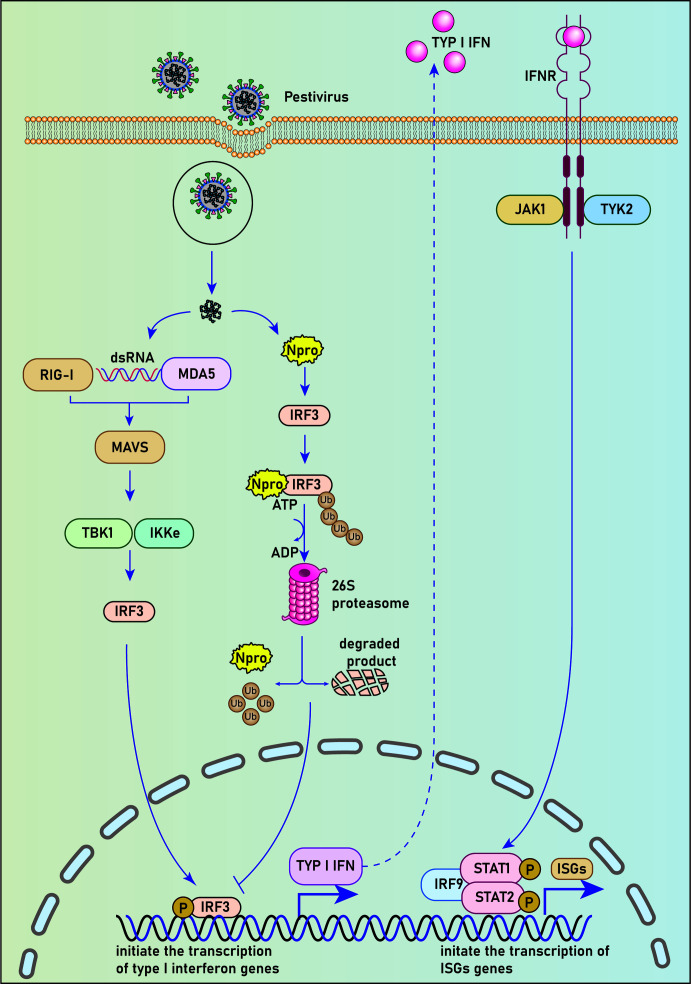
N^pro^ blocks the host’s IFN-activated immune response by degradation of IRF3. Upon viral infection, pathogenic associated molecular patterns (PAMPs) are recognized by cellular pattern recognition receptors (PRRs). A series of cellular pathways were activated subsequently to promote the translocation of phosphorylated IRF3 into the nucleus and initiate the transcription of type I interferon genes by binding to IFN-α/β promoters. N^pro^ could interact with IRF3 before it’s phosphorylation-induced activation, leading to the ubiquitination and proteasomal degradation of IRF-3 and subsequent inhibition of the type I interferon response.

By luciferase reporter gene experiment, La Rocca et al. found that CSFV-infected cells could inhibit IRF3 gene transcription. The use of cell lines expressing CSFV N^pro^ confirmed that the N^pro^ protein reduced the expression of IRF3, suggesting that this single viral protein specific to the pestiviruses can inhibit interferon production in the innate immune response to the virus (37) (**Figure 1**). Hilton et al. reported that NCP-BVDV (pe515) infection could induce the translocation of a small amount of IRF-3 from the cytoplasm to the nucleus at the early stage of infection. In addition, most IRF3 in the cytoplasm was degraded by the ubiquitination-proteasome pathway mediated by the N^pro^ protein (27). Similar to the NCP-BVDV virus, CP-BVDV (NADL strain) does not induce interferon response after infection and blocks interferon-stimulating genes induced by paramyxovirus infection, resulting in a significant decrease in IRF3 expression. However, the IRF3 repression activity is considered independent of the protease activity of N^pro^. Further studies revealed that N^pro^ could interact with IRF3 before its phosphorylation-induced activation, leading to the ubiquitination and proteasomal degradation of IRF3 (38).

The N^pro^ protein of CSFV can also mediate the degradation of IRF3 after interaction with it. However, different from the research results of La Rocca et al., There are other studies reported that CSFV infection does not inhibit a cytomegalovirus (CMV) promoter-driven IRF3 expression. Furthermore, CSFV neither reduces the transcriptional activity of the IRF3 promoter nor affects the stability of IRF3 mRNA (39, 40).

Ubiquitin contains 76 amino acid residues and is highly conserved in all eukaryotes (41). Selective binding of linear ubiquitin to a protein is the initial signal for target protein degradation. Ubiquitin chains can be conjugated to the specific protein substrate through an isopeptide bond between the ubiquitin C-terminal glycine residues and the lysine residues in the substrate. Proteasome-dependent degradation of proteins mediated by ubiquitination regulates a variety of biological reactions in the body, including cell cycle, signaling, DNA repair, and apoptosis (42, 43).

The ubiquitin modification process requires successive events associated with three enzymes: an E1 ubiquitin-activating enzyme, an E2 ubiquitin-conjugating enzyme, and an E3 ubiquitin ligase. Ubiquitin is first connected to the catalytic cysteine residues of endogenous E1 protein by an intermediate thioester bond. It is then conveyed to an E2 conjugating enzyme through a trans-esterification reaction. Subsequently, E3 ubiquitin ligase transfers the activated ubiquitin from an E2 to substrates. Finally, the substrate labeled with ubiquitin is delivered to the 26S proteasome for degradation (44). Once the E1 ubiquitin-activating enzyme was thermal inactivated, the degradation activity of IRF3 mediated by the N^pro^ protein of CP-BVDV was lost (39), indicating that the E1 ubiquitin-activating enzyme is involved in the N^pro^ protein-mediated ubiquitin modification and degradation of IRF3.

Besides contributing to the antiviral immune response, IRF3 participates in the control of the cell cycle, apoptosis, and tumor suppression as well (45). When cells are infected with Sendai virus or stimulated by double-stranded RNA, IRF3 can bind to cytoplasmic Bax *via* its BH3 region and transfer Bax to mitochondria to activate apoptosis. It has been shown that wild-type N^pro^ could restrain apoptosis signals induced by astrosporin, interferon, double-stranded RNA, sodium arsenate, and hydrogen peroxide, which was mainly achieved by the degradation of IRF3 protein. In addition, the N^pro^ protein can inhibit the Bax-dependent apoptosis pathway by inhibiting mitochondrial lysis and Bax redistribution through interaction with the mitochondrial lumen. Moreover, the N^pro^ protein could rapidly localize to ubiquitin-containing peroxisome. Thus, the N^pro^ protein may bind to IRF3 and degrade it by transporting it to ubiquitin-containing peroxisome (46).

Peptide-prolyl isomerase (Pin1), Ro52 (TRIM21), RBCC protein interacting with PKC1 (RBCK1), RTA-associated ubiquitin ligase (RAUL), Tripartite motif 21 (TRIM21), Forkhead transcription factors of the O class (FOXO1), Casitas B-lineage lymphoma (c-Cbl) have been suggested as E3 ligases to invoke ubiquitination and subsequent degradation of IRF3 in the cytoplasm, thus inhibiting the production of type I IFN (47–51). However, it is unclear which E3 ubiquitin ligase contributes to the ubiquitination degradation of IRF3 mediated by the N^pro^ protein. It is worthy of further exploration whether N^pro^ has crosstalk with these E3 ligases or molecules to regulate the ubiquitination and subsequent degradation of IRF3.

Currently, the N^pro^ binding site on IRF3 is unclear. It has been revealed that N^pro^ can interact with IRF3 directly and forms a soluble 1:1 complex by utilizing recombinant N^pro^ and IRF3 proteins. This interaction requires the complete IRF3 protein rather than any of the single domains, the DBD in N-terminal or the IAD in C-terminal (52). It has been also shown in a previous mammalian two-hybrid analysis that the association of N^pro^ with IRF3 needs both the DBD and the IAD of IRF3 (53). However, the exact arrangement of DBD and the IAD in the IRF3 monomer and dimer is still unknown (35, 54, 55). As the ~60-amino-acid linker region between the two domains is somewhat helical but not structured in the absence of either DBD or IAD (56). The intact linker is thus suggested to be involved in N^pro^ binding (52). N^pro^ has been shown to interact with the IRF3 monomer and phosphomimetic dimer, indicating that the N^pro^ binding site on IRF3 contains areas not affected by the phosphorylation and subsequent activation status of IRF3 (52). N^pro^ can also interact with IRF3 in the complex with its transcriptional cofactor, the CREB-binding protein (CBP). Therefore, the contact surface in the IRF3 dimer and CBP binding site is not required for N^pro^ binding (52).

BVDV N^pro^ protein has been shown to degrade IRF3 in the cytoplasm, whereas IRF3 in the nucleus is resistant to this degradation (26). The influence of cellular localization of N^pro^ on IRF3 degradation is unclear. A recombinant virus vSMS-IRF3 was constructed by inserting the IRF3 gene sequence between the 13th and 14th amino acid sites of the N^pro^ protein of the highly virulent CSFV Shimen strain by Li et, al (57). The fusion protein of IRF3-N^pro^ expressed by the recombinant virus only located in the cytoplasm and vSMS-IRF3 was significantly attenuated. Pigs inoculated with the recombinant virus were all resistant to the lethal CSFV challenge, but the parent virus showed a typical virulent phenotype (57). Therefore, it was suggested that the nuclear localization of N^pro^ is essential to the replication and virulence of CSFV (57).

However, a previous study showed that any mutants of L8P, E22L, and H49V in N^pro^ could abolish its IFN-α/β antagonistic activity, revealing that the 49 amino acids in the N-terminal of N^pro^ protein are necessary to type-I IFN suppression (33). Based on this, we speculate that the insertion of the IRF3 gene into the N^pro^ gene may eliminate its function of IRF3 degradation. Thus, the attenuation of vSM-IRF3 may be caused by the loss of the IFN-α/β antagonistic activity of IRF3-N^pro^ rather than its cytoplasmic localization. Moreover, N^pro^ was observed in the nucleus in a diffuse manner (58, 59), and could bind to IRF3 dimer or the IRF3 dimer in the complex with CBP (52). Therefore, the insertion of IRF3 into N^pro^ could also affect the cellular diffusion of the IRF3-N^pro^ fusion protein, leading to its accumulation in the cytoplasm. The effects of cellular localization of N^pro^ protein on CSFV virulence need further study.

Whether N^pro^ protein is the main determinant of the virulence of pestiviruses is still a controversial topic. Continuous passage of CSFV attenuated vaccine strain GPE- in pigs restored its virulence, but did not regain the ability of its N^pro^ to degrade IRF3 (60). However, strains containing the N136D mutation in N^pro^ restored the IRF3 degradation activity and IFN-α/β antagonistic ability *in vitro* as well as pathogenicity *in vivo*. These results demonstrate that the N^pro^ protein makes a decisive contribution to the virulence of pestiviruses, but there are other factors that can regulate the virulence of pestiviruses.

The N^pro^ protein of CSFV can also interact with IRF7 in plasmacytoid dendritic cells, down-regulating the expression level of IRF7 protein and further inhibiting the IFN-α expression. Whereas, the molecular mechanism of N^pro^ inhibiting the expression of IRF7 protein is still unclear. It is certain, however, that this antagonism does not involve either polyubiquitination or protease degradation pathways (53).

### The zinc atom binding motif of N^pro^ is critical for the degradation of IRF3

Analysis by sequence alignment revealed that the C-terminal half of the N^pro^ protein contains a conserved metal binding TRASH motif composed of Cys-X21-Cys-X3-Cys (where X is any amino acid). TRASH motif commonly exists in proteins associated with heavy metal recognizing, resistance, transcription regulation, cation transportation, and hydrogenase. Inductively coupled plasma–mass spectrometry (ICP-MS) assay indicated that each N^pro^ protein molecule could coordinate a single zinc atom. Site-directed mutagenesis studies revealed that the zinc-binding sites of N^pro^ protein include Cys112, Cys134, Cys138, and probably Asp136. These zinc-binding site mutations lead to the deficit of N^pro^ protein-mediated IRF3 degradation in cells inoculated with CSFV, suggesting that the zinc-binding capacity of N^pro^ protein is critical for virus-mediated IRF3 degradation (61). In addition, the zinc-binding domain of N^pro^ is critical for its protein stability and its interaction with IRF7 (62).

## N^pro^ interacts extensively with host proteins

### Proteins in cytoplasmic ribonucleoprotein particles

Recently, pull-down combined mass spectrometry showed that N^pro^ binds to more than 55 kinds of proteins, mainly RNA helicase A (DHX9), Y-box binding protein (YBX1), DDX3, DDX5, IGF2BP1, eIF3, and other ribosomal proteins, multiple myeloma tumor protein 2, interleukin enhancer binding factor 3 (IEBP3) guanine nucleotide-binding protein 3, and polyadenylate-binding protein 1 (PABP-1). Many of the interacting proteins are components in cytoplasmic ribonucleoprotein particles (RNPs). They play roles in regulating the translation of mRNA and could be recruited into stress granules to regulate the translational initiation rate or mRNA degradation (63). The assembled stress granules might control the proliferation of viruses and some viruses could in turn hinder their formation or even disassemble them (64). It has been suggested that N^pro^ could redistribute to stress granules after interaction with YBX1 through its TRASH domain. When exposed to oxidative stress, cells expressing N^pro^ alone assembled stress granules and N^pro^ colocalized with stress granule proteins. In contrast, the formation of stress granules in NCP-BVDV-infected cells was inhibited, indicating that this inhibition was not caused by N^pro^ binding to ribonucleoproteins (63). Thereby, N^pro^ may not influence the function and location of ribonucleoproteins although it could be localized to stress granules by interacting with these host proteins. As some interacting proteins of N^pro^ are also involved in RISC function during RNA silencing, further studies were conducted to determine whether N^pro^ affected RNA interference (RNAi). However, the outcomes suggested the expression of N^pro^ had no influence on RNAi silencing activity (65).

### S100A9

S100A9, one of the danger-associated molecular patterns (DAMPs) proteins, is vital in the innate immune system and always accumulates in large amounts in ectocytic space during inflammation responses (66). Additionally, the S100A9 could effectively trigger inflammatory responses through Toll-like receptor 4 (TLR4) as a homodimer (67–73). In the heterodimer with S100A8, the S100A9 exerts antimicrobial activity by inhibiting microbes from acquiring nutrients (74–79). Yet, the function of S100A9 in virus infection is unclear. It has been shown that the amount of S100A9 was increased after treatment with poly (I: C), which is an analog of viral dsRNA (80). The expression of S100A9 is also highly enhanced in human papillomavirus-associated dysplastic tissues (81) and BKV-infected recipients post-kidney transplantation (82). Likewise, high levels of S100A9 expression were observed in lungs and livers in autopsied subjects with COVID-19 and pre-existing chronic liver disease (83). Darweesh et al. reported that NCP-BVDV2a 1373 N^pro^ protein has a strong interaction with cellular S100A9 protein. Furthermore, the N^pro^ protein enhances the replication of BVDV in infected cells by inhibiting S100A9 activity in epidermal cells (84).

### TRIM56

Currently, the TRIM family consists of more than 60 members and could be divided into 11 subfamilies (85). Although their exact functions are still unclear, the TRIM proteins contribute to a large variety of biological activities, such as cell proliferation, development, differentiation, immunity, apoptosis, and innate immune response to pathogens (86–90). As a protein of the fifth subfamily of TRIM, TRIM56 is expressed in the cytoplasm after type I interferon stimulation (91, 92). A previous study has identified TRIM56 as a cellular protein that associates with BVDV N^pro^ through its C-terminal portion. Although TRIM56 has RING-dependent E3 ubiquitin ligase function, it is not involved in N^pro^-mediated IRF3 degradation nor degrade N^pro^. Furthermore, it was suggested that both ectopically and endogenously expressed TRIM56 contribute to impaired replication of BVDV due to its E3 ligase activity. In contrast, the downregulation of TRIM56 expression largely improved BVDV proliferation. Moreover, it is the integrity of the TRIM56 C-terminal, rather than the TRIM56-N^pro^ association that contributes to TRIM56’s antiviral activity (91).

### pIκBα

To discover host proteins that could bind to N^pro^ of CSFV, Doceul, et al. (58) conducted a yeast two-hybrid assay of a human library. It was revealed that N^pro^ had a direct association with IκBα, which is responsible for apoptosis regulation, the immune reaction, and IFN expression. As an inhibitor of NF-κB, IκBα is also a prime target for immune evasion strategies developed by many viruses (93–95). Further studies suggested the interaction of N^pro^ with aa 213-317 of the C-terminus of pIκBα (pig IκBα) (58), which also contact with NF-κB through the domain between aa214-280 (96, 97). This suggests that N^pro^ competes with NF-κB for unbound pIκBα (58).

Before stimulation, NF-κB remains in an inactive state in the cytoplasm due to its interaction with IκBα, which covers the nuclear localization signals of NF-κB. It has been reported that NF-κB/IκBα complex could be triggered by phosphorylation upon various stimulation, such as viruses and bacteria (98–101). In this case, IκBα is phosphorylated at Ser32 and Ser36 by the IKKβ subunit following the activation of the IKK complex (IKKα/IKKβ/IKKγ). Then, the E3 ubiquitin ligase complex, SCFβ−TRCP, ubiquitinates IκBα and targets it for degradation by the 26S proteasome, resulting in the release of NF-κB for nuclear translocation (102–105). However, the activated NF-κB initiates regeneration of IκBα, which detaches NF-κB from DNA after its translocation to the nucleus, and conveys NF-κB to the cytoplasm in a nuclear export sequence-dependent process (106–108). Tumor necrosis factor-alpha receptor (TNFR) activated by binding with TNF-α is one of the principal receptors that mediate NF-κB activation (109).

**Figure 2:**
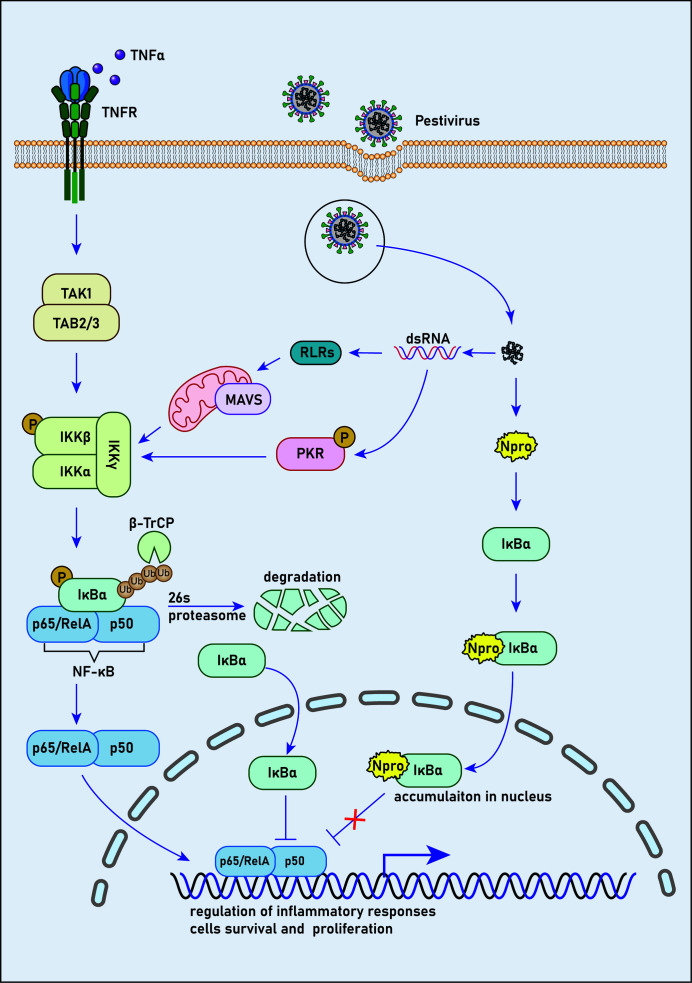
N competes with NF-κB to bind with IκBα. Prior to stimulation, NF-κB remains an inactive state in the cytoplasm due to its interaction with IκBα, which masks the unclear localization signals of NF-κB. NF-κB/IκBα complex is activated by phosphorylation in response to various stimuli, such as viral and bacterial pathogens. In this case, IκBα is phosphorylated at Ser32 and Ser36 by the IKKβ subunit following the activation of IKK complex. Then, the E3 ubiquitin ligase complex, SCFβ−TRCP, ubiquitinates IκBα and targets it for degradation by the 26S proteasome, leading to the release of NF-κB for nuclear translocation. However, NF-κB activation induces rapid resynthesis of IκBα, which translocates to the nucleus, dissociates NF-κB from DNA and transports NF-κB to the cytoplasm in a nuclear export sequence-dependent process.

It’s reasonable to speculate that under stimulation of TNF-α, new synthesized cytoplasmic IκBα induced by NF-κB activation could bind to N^pro^ in cells expressing N^pro^ proteins. Therefore, limited unbound IκBα translocate into the nucleus, and thus the suppression of NF-κB DNA-binding activity by IκBα should be restricted (**Figure 2**). It has been suggested that HIV-1 tat transactivator could activate NF-κB by interacting with IκBα and by inhibiting the repressor from binding to the NF-κB complex (110). However, the ability of the p65 subunit of NF-κB to bind the promoter sequence in CSFV-infected PK15 cells was not affected by functional analysis (111). Furthermore, after TNF-α stimulation of N^pro^ stable expression PK15 cells, a high concentration of pIκBα was observed in the nucleus, but the function and expression of NF-κB did not change significantly (58). Therefore, TNF-α may stimulate the rapid resynthesis and massive nuclear translocation of pIκBα, many of which are bound to N^pro^ and does not affect the action of NF-κB thus resulting in the accumulation of a large amount of pIκBα in the nucleus. However, the effect of N^pro^ binding to pIκBα on the activity of NF-κB in the nucleus and cytoplasm is worth further investigation.

### HAX-1

HS-1-associated protein X-1 (HAX-1), a protein of approximately 35-kDa, is universally synthesized in murine and human tissues (112, 113), especially in the mitochondria (114). Primarily, it was shown to play a role in the control of apoptosis or programmed cell death (114). A recent study has indicated that HAX-1 may also contribute to the control of calcium homeostasis and cell survival in cardiac tissue (115). It was found that the protein could bind to the N^pro^ protein of CSFV by yeast two-hybrid, and the interaction between the two proteins was further confirmed by co-immunoprecipitation assay (116). During CSFV infection, the expression level of HAX-1 did not change significantly, indicating that N^pro^ interacting with HAX-1 could not degrade it. However, in the cells co-transfected with HAX-1 and N^pro^, the two proteins were significantly transported to the endoplasmic reticulum, and the N^pro^ protein in the nucleus was significantly reduced (116). Significantly, the relocation of HAX-1 to the ER in the presence of phospholamban (PLN), a crucial regulator of Ca^2+^ homeostasis and contractility in the heart, correlated with stronger resistance to apoptosis (117). Therefore, it could be hypothesized that during CSFV infection, N^pro^ protein may bind to HAX-1 in the cytoplasm and transport it to ER to enhance the tolerance of infected cells to apoptosis. Thus, less N^pro^ protein synthesized in the cytoplasm diffuses into the nucleus. However, further research is needed to confirm this hypothesis.

### PCBPs

As members of the K homology (KH) domain superfamily, Poly(C)-binding proteins (PCBPs) are known for their interaction with both RNA and DNA specifically. It has been suggested that the KH domain superfamily proteins associate with the stability of cellular mRNAs (118–120), regulate their translation (121–125), and also involve in the host antiviral reaction (126, 127). Among the PCBPs, PCBP1 is an ssDNA-binding protein that contributes to the transcription of the neuronal μ-opioid receptor gene (122). Cytoplasmic and nuclear expression of CBP1 been demonstrated (128). Li et al. found that PCBP1 protein could interact with N^pro^ protein. Knocking down the expression of PCBP1 could inhibit the replication of CSFV, while overexpression of PCBP1 could promote the reproduction of CSFV. PCBP1 inhibits the IFN signaling pathway by degrading MAVS and enhances the replication of CSFV genomic RNA, thus promoting the proliferation of CSFV (129). However, whether the interaction between PCBP1 and N^pro^ has any effect on their cellular localization or CSFV replication needs further exploration.

## Response of N^pro^ protein to type-3 interferon

N^pro^ has also been suggested to inhibit the innate immune reaction by restricting type III IFNs (IFN-λs). Although many similarities exist between types I and III IFNs in the signaling networks and expression processes (130, 131), a host of distinctions are evident. Different from type I IFNs induction which needs all IFN enhanceosome elements, type III IFNs are induced independent of IRFs or NF-κB (132). Besides, unlike the ubiquitous receptors for type I IFNs, type III IFN receptors are mainly expressed in mucosal epithelia (133). Thus, type III IFNs are considered to be crucial for immune responses in the respiratory and gastrointestinal tracts (134). As IRFs and NF-κB are important regulators for type III IFNs, IRF1 may have a specific function in this process (135). N^pro^-deficient CSFV has been shown to significantly enhance the transcription level of IFN-λs 24h post-infection. In contrast, overexpression of N^pro^ significantly reduced the IFN-λs transcription and IFN-λ3 promoter activity. Moreover, in poly (I: C)-treated IPEC-J2 cells, overexpression of N^pro^ or infection with wtCSFV not only down-regulated the production and the promoter activity of IRF1 significantly but also inhibited IRF1 nuclear translocation. This suggests that N^pro^ could restrict type III IFNs response by preventing the production and nuclear translocation of IRF1 (134).

## Conclusion

Pestiviruses are counted among the highly destructive and economically important pathogens, which have evolved many strategies to evade elimination by the host antiviral immune response. Studies emphasizing various molecular techniques undertaken during the last two decades have elucidated at least two viral proteins (N^pro^ and E^rns^ RNase) as IFN antagonists of pestiviruses. Unlike repression of the interferon response *via* the effect of N^pro^ on IRF3, the secreted E^rns^ protein distributed by the bloodstream could be taken up by cells, specifically PDCs, and degrade pestiviral RNA. Therefore, E^rns^ hinders IFN production induced by the extracellular synthetic or viral ss- or dsRNAs (136–138). Thus, we conclude that pestiviruses evade the host’s IFN-activated innate antiviral immune response in a complex way to establish and maintain a persistent infection status. This article mainly reviewed the progress of innate immune evasion mediated by N^pro^ of pestiviruses. while its detailed process concerning blocking the IFN-1 response remains obscure. Further understanding of the approaches employed by viruses of this genus to control immune response to escape the innate immune system is in need, which will eventually contribute to developing effective strategies to prevent and control pestivirus infection.

## Author contributions

Writing-original draft preparation: SW and XLi. Writing review: YS, XLv, and KL. Figures: SW. Supervision: JZ. Funding acquisition: SW, YS, JR, and JZ. All authors contributed to the article and approved the submitted version.

## References

[1] Houe H. Economic impact of BVDV infection in dairies. Biologicals (2003) 31:137–43. doi: 10.1016/s1045-1056(03)00030-7 12770546

[2] Moennig V Becher P. Pestivirus control programs: How far have we come and where are we going? Anim Health Res Rev (2015) 16:83–7. doi: 10.1017/s1466252315000092 26050577

[3] Smith DB Meyers G Bukh J Gould EA Monath T Scott Muerhoff A. Proposed revision to the taxonomy of the genus pestivirus, family flaviviridae. J Gen Virol (2017) 98:2106–12. doi: 10.1099/jgv.0.000873 PMC5656787 28786787

[4] Schweizer M Peterhans E. Pestiviruses. Annu Rev Anim Biosci (2014) 2:141–63. doi: 10.1146/annurev-animal-022513-114209 25384138

[5] Nettleton PF Gilray JA Russo P Dlissi E. Border disease of sheep and goats. Vet Res (1998) 29:327–40. 9689745

[6] Potgieter LN. Bovine respiratory tract disease caused by bovine viral diarrhea virus. Vet Clin North Am Food Anim Pract (1997) 13:471–81. doi: 10.1016/s0749-0720(15)30309-1 9368990

[7] Lanyon SR Hill FI Reichel MP Brownlie J. Bovine viral diarrhoea: Pathogenesis and diagnosis. Vet J (2014) 199:201–9. doi: 10.1016/j.tvjl.2013.07.024 24053990

[8] Postel A Hansmann F Baechlein C Fischer N Alawi M Grundhoff A. Presence of atypical porcine pestivirus (APPV) genomes in newborn piglets correlates with congenital tremor. Sci Rep (2016) 6:27735. doi: 10.1038/srep27735 27292119 PMC4904412

[9] Postel A Schmeiser S Oguzoglu TC Indenbirken D Alawi M Fischer N. Close relationship of ruminant pestiviruses and classical swine fever virus. Emerg Infect Dis (2015) 21:668–72. doi: 10.3201/eid2104.141441 PMC4378490 25811683

[10] Hause BM Collin EA Peddireddi L Yuan F Chen Z Hesse RA. Discovery of a novel putative atypical porcine pestivirus in pigs in the USA. J Gen Virol (2015) 96:2994–8. doi: 10.1099/jgv.0.000251 26219947

[11] Kirkland PD Frost MJ Finlaison DS King KR Ridpath JF Gu X. Identification of a novel virus in pigs–bungowannah virus: A possible new species of pestivirus. Virus Res (2007) 129:26–34. doi: 10.1016/j.virusres.2007.05.002 17561301

[12] Liu L Kampa J Belák S Baule C. Virus recovery and full-length sequence analysis of atypical bovine pestivirus Th/04_KhonKaen. Vet Microbiol (2009) 138:62–8. doi: 10.1016/j.vetmic.2009.03.006 19349128

[13] Schirrmeier H Strebelow G Depner K Hoffmann B Beer M. Genetic and antigenic characterization of an atypical pestivirus isolate, a putative member of a novel pestivirus species. J Gen Virol (2004) 85:3647–52. doi: 10.1099/vir.0.80238-0 15557237

[14] Shi K Xie S Sun W Liu H Zhao J Yin Y. Evolution and genetic diversity of atypical porcine pestivirus (APPV) from piglets with congenital tremor in guangxi province, southern China. Vet Med Sci (2021) 7:714–23. doi: 10.1002/vms3.407 PMC8136935 33314734

[15] Şevik M. Genomic characterization of pestiviruses isolated from bovine, ovine and caprine foetuses in Turkey: A potentially new genotype of pestivirus I species. Transbound Emerg Dis (2021) 68:417–26. doi: 10.1111/tbed.13691 32564510

[16] Chen M Liu M Liu S Shang Y. HoBi-like pestivirus infection leads to bovine death and severe respiratory disease in China. Transbound Emerg Dis (2021) 68:1069–74. doi: 10.1111/tbed.13832 32926568

[17] Pan S Yan Y Shi K Wang M Mou C Chen Z. Molecular characterization of two novel atypical porcine pestivirus (APPV) strains from piglets with congenital tremor in China. Transbound Emerg Dis (2019) 66:35–42. doi: 10.1111/tbed.13029 30281923

[18] Becher P Orlich M Shannon AD Horner G König M Thiel HJ. Phylogenetic analysis of pestiviruses from domestic and wild ruminants. J Gen Virol (1997) 78(Pt 6):1357–66. doi: 10.1099/0022-1317-78-6-1357 9191930

[19] Avalos-Ramirez R Orlich M Thiel HJ Becher P. Evidence for the presence of two novel pestivirus species. Virology (2001) 286:456–65. doi: 10.1006/viro.2001.1001 11485413

[20] Vilcek S Ridpath JF Van Campen H Cavender JL Warg J. Characterization of a novel pestivirus originating from a pronghorn antelope. Virus Res (2005) 108:187–93. doi: 10.1016/j.virusres.2004.09.010 15681069

[21] Neill JD Ridpath JF Fischer N Grundhoff A Postel A Becher P. Complete genome sequence of pronghorn virus, a pestivirus. Genome Announc (2014) 2(3):e00575-14. doi: 10.1128/genomeA.00575-14 24926058 PMC4056301

[22] Tao J Liao J Wang Y Zhang X Wang J Zhu G. Bovine viral diarrhea virus (BVDV) infections in pigs. Vet Microbiol (2013) 165:185–9. doi: 10.1016/j.vetmic.2013.03.010 23587625

[23] Tautz N Tews BA Meyers G. The molecular biology of pestiviruses. Adv Virus Res (2015) 93:47–160. doi: 10.1016/bs.aivir.2015.03.002 26111586

[24] Tratschin JD Moser C Ruggli N Hofmann MA. Classical swine fever virus leader proteinase npro is not required for viral replication in cell culture. J Virol (1998) 72:7681–4. doi: 10.1128/jvi.72.9.7681-7684.1998 PMC110041 9696875

[25] Schweizer M Peterhans E. Noncytopathic bovine viral diarrhea virus inhibits double-stranded RNA-induced apoptosis and interferon synthesis. J Virol (2001) 75:4692–8. doi: 10.1128/jvi.75.10.4692-4698.2001 PMC114223 11312340

[26] Hilton L Moganeradj K Zhang G Chen YH Randall RE McCauley JW. The NPro product of bovine viral diarrhea virus inhibits DNA binding by interferon regulatory factor 3 and targets it for proteasomal degradation. J Virol (2006) 80:11723–32. doi: 10.1128/jvi.01145-06 PMC1642611 16971436

[27] Tao J Liao J Wang J Zhang X Zhang Q Zhu L. Pig BVDV-2 non-structural protein (N(pro)) links to cellular antiviral response *in vitro* . Virus Genes (2017) 53:233–9. doi: 10.1007/s11262-016-1410-2 27866318

[28] Ruggli N Tratschin JD Schweizer M McCullough KC Hofmann MA Summerfield A. Classical swine fever virus interferes with cellular antiviral defense: Evidence for a novel function of n(pro). J Virol (2003) 77:7645–54. doi: 10.1128/jvi.77.13.7645-7654.2003 PMC164809 12805464

[29] Ruggli N Bird BH Liu L Bauhofer O Tratschin JD Hofmann MA. N(pro) of classical swine fever virus is an antagonist of double-stranded RNA-mediated apoptosis and IFN-alpha/beta induction. Virology (2005) 340:265–76. doi: 10.1016/j.virol.2005.06.033 16043207

[30] Bauhofer O Summerfield A McCullough KC Ruggli N. Role of double-stranded RNA and npro of classical swine fever virus in the activation of monocyte-derived dendritic cells. Virology (2005) 343:93–105. doi: 10.1016/j.virol.2005.08.016 16154171

[31] Mou C Pan S Wu H Chen Z. Disruption of interferon-β production by the n(pro) of atypical porcine pestivirus. Virulence (2021) 12:654–65. doi: 10.1080/21505594.2021.1880773 PMC7872032 33538238

[32] Richter M König P Reimann I Beer M. N pro of bungowannah virus exhibits the same antagonistic function in the IFN induction pathway than that of other classical pestiviruses. Vet Microbiol (2014) 168:340–7. doi: 10.1016/j.vetmic.2013.11.038 24398226

[33] Gil LH Ansari IH Vassilev V Liang D Lai VC Zhong W. The amino-terminal domain of bovine viral diarrhea virus npro protein is necessary for alpha/beta interferon antagonism. J Virol (2006) 80:900–11. doi: 10.1128/jvi.80.2.900-911.2006 PMC1346884 16378992

[34] Hiscott J. Triggering the innate antiviral response through IRF-3 activation. J Biol Chem (2007) 282:15325–9. doi: 10.1074/jbc.R700002200 17395583

[35] Qin BY Liu C Lam SS Srinath H Delston R Correia JJ. Crystal structure of IRF-3 reveals mechanism of autoinhibition and virus-induced phosphoactivation. Nat Struct Biol (2003) 10:913–21. doi: 10.1038/nsb1002 14555996

[36] Honda K Taniguchi T. IRFs: Master regulators of signalling by toll-like receptors and cytosolic pattern-recognition receptors. Nat Rev Immunol (2006) 6:644–58. doi: 10.1038/nri1900 16932750

[37] Baigent SJ Goodbourn S McCauley JW. Differential activation of interferon regulatory factors-3 and -7 by non-cytopathogenic and cytopathogenic bovine viral diarrhoea virus. Vet Immunol Immunopathol (2004) 100:135–44. doi: 10.1016/j.vetimm.2004.04.003 15207451

[38] La Rocca SA Herbert RJ Crooke H Drew TW Wileman TE Powell PP. Loss of interferon regulatory factor 3 in cells infected with classical swine fever virus involves the n-terminal protease, npro. J Virol (2005) 79:7239–47. doi: 10.1128/jvi.79.11.7239-7247.2005 PMC1112113 15890962

[39] Chen Z Rijnbrand R Jangra RK Devaraj SG Qu L Ma Y. Ubiquitination and proteasomal degradation of interferon regulatory factor-3 induced by npro from a cytopathic bovine viral diarrhea virus. Virology (2007) 366:277–92. doi: 10.1016/j.virol.2007.04.023 PMC2000802 17531282

[40] Bauhofer O Summerfield A Sakoda Y Tratschin JD Hofmann MA Ruggli N. Classical swine fever virus npro interacts with interferon regulatory factor 3 and induces its proteasomal degradation. J Virol (2007) 81:3087–96. doi: 10.1128/jvi.02032-06 PMC1866024 17215286

[41] Seago J Hilton L Reid E Doceul V Jeyatheesan J Moganeradj K. The npro product of classical swine fever virus and bovine viral diarrhea virus uses a conserved mechanism to target interferon regulatory factor-3. J Gen Virol (2007) 88:3002–6. doi: 10.1099/vir.0.82934-0 17947522

[42] Nandi D Tahiliani P Kumar A Chandu D. The ubiquitin-proteasome system. J Biosci (2006) 31:137–55. doi: 10.1007/bf02705243 16595883

[43] Wang J Maldonado MA. The ubiquitin-proteasome system and its role in inflammatory and autoimmune diseases. Cell Mol Immunol (2006) 3:255–61. 16978533

[44] Liu YC. Ubiquitin ligases and the immune response. Annu Rev Immunol (2004) 22:81–127. doi: 10.1146/annurev.immunol.22.012703.104813 15032575

[45] Honda K Takaoka A Taniguchi T. Type I interferon [corrected] gene induction by the interferon regulatory factor family of transcription factors. Immunity (2006) 25:349–60. doi: 10.1016/j.immuni.2006.08.009 16979567

[46] Jefferson M Whelband M Mohorianu I Powell PP. The pestivirus n terminal protease n(pro) redistributes to mitochondria and peroxisomes suggesting new sites for regulation of IRF3 by n(pro.). PloS One (2014) 9:e88838. doi: 10.1371/journal.pone.0088838 24551175 PMC3925175

[47] Yu Y Hayward GS. The ubiquitin E3 ligase RAUL negatively regulates type i interferon through ubiquitination of the transcription factors IRF7 and IRF3. Immunity (2010) 33:863–77. doi: 10.1016/j.immuni.2010.11.027 PMC3012379 21167755

[48] Lei CQ Zhang Y Xia T Jiang LQ Zhong B Shu HB. FoxO1 negatively regulates cellular antiviral response by promoting degradation of IRF3. J Biol Chem (2013) 288:12596–604. doi: 10.1074/jbc.M112.444794 PMC3642307 23532851

[49] Zhao X Zhu H Yu J Li H Ge J Chen W. C-cbl-mediated ubiquitination of IRF3 negatively regulates IFN-β production and cellular antiviral response. Cell Signal (2016) 28:1683–93. doi: 10.1016/j.cellsig.2016.08.002 27503123

[50] Higgs R NG J Ben Larbi N Breen EP Fitzgerald KA Jefferies CA. The E3 ubiquitin ligase Ro52 negatively regulates IFN-beta production post-pathogen recognition by polyubiquitin-mediated degradation of IRF3. J Immunol (2008) 181:1780–6. doi: 10.4049/jimmunol.181.3.1780 PMC2824853 18641315

[51] Saitoh T Tun-Kyi A Ryo A Yamamoto M Finn G Fujita T. Negative regulation of interferon-regulatory factor 3-dependent innate antiviral response by the prolyl isomerase Pin1. Nat Immunol (2006) 7:598–605. doi: 10.1038/ni1347 16699525

[52] Gottipati K Holthauzen LM Ruggli N Choi KH. Pestivirus npro directly interacts with interferon regulatory factor 3 monomer and dimer. J Virol (2016) 90:7740–7. doi: 10.1128/jvi.00318-16 PMC4988160 27334592

[53] Fiebach AR Guzylack-Piriou L Python S Summerfield A Ruggli N. Classical swine fever virus n(pro) limits type I interferon induction in plasmacytoid dendritic cells by interacting with interferon regulatory factor 7. J Virol (2011) 85:8002–11. doi: 10.1128/jvi.00330-11 PMC3147952 21680532

[54] Takahasi K Suzuki NN Horiuchi M Mori M Suhara W Okabe Y. X-Ray crystal structure of IRF-3 and its functional implications. Nat Struct Biol (2003) 10:922–7. doi: 10.1038/nsb1001 14555995

[55] Fujii Y Shimizu T Kusumoto M Kyogoku Y Taniguchi T Hakoshima T. Crystal structure of an IRF-DNA complex reveals novel DNA recognition and cooperative binding to a tandem repeat of core sequences. EMBO J (1999) 18:5028–41. doi: 10.1093/emboj/18.18.5028 PMC1171574 10487755

[56] Shukla H Vaitiekunas P Majumdar AK Dragan AI Dimitriadis EK Kotova S. The linker of the interferon response factor 3 transcription factor is not unfolded. Biochemistry (2012) 51:6320–7. doi: 10.1021/bi300260s 22812703

[57] Li Y Shen L Sun Y Wang X Li C Huang J. Effects of the nuclear localization of the n(pro) protein of classical swine fever virus on its virulence in pigs. Vet Microbiol (2014) 174:391–8. doi: 10.1016/j.vetmic.2014.09.027 25457365

[58] Doceul V Charleston B Crooke H Reid E Powell PP Seago J. The npro product of classical swine fever virus interacts with IkappaBalpha, the NF-kappaB inhibitor. J Gen Virol (2008) 89:1881–9. doi: 10.1099/vir.0.83643-0 18632959

[59] Li Y Shen L Li C Huang J Zhao B Sun Y. Visualization of the npro protein in living cells using biarsenically labeling tetracysteine-tagged classical swine fever virus. Virus Res (2014) 189:67–74. doi: 10.1016/j.virusres.2014.04.018 24815879

[60] Tamura T Nagashima N Ruggli N Summerfield A Kida H Sakoda Y. Npro of classical swine fever virus contributes to pathogenicity in pigs by preventing type I interferon induction at local replication sites. Vet Res (2014) 45:47. doi: 10.1186/1297-9716-45-47 24742209 PMC4018971

[61] Szymanski MR Fiebach AR Tratschin JD Gut M Ramanujam VM Gottipati K. Zinc binding in pestivirus n(pro) is required for interferon regulatory factor 3 interaction and degradation. J Mol Biol (2009) 391:438–49. doi: 10.1016/j.jmb.2009.06.040 19540847

[62] Zögg T Sponring M Schindler S Koll M Schneider R Brandstetter H. Crystal structures of the viral protease npro imply distinct roles for the catalytic water in catalysis. Structure (2013) 21:929–38. doi: 10.1016/j.str.2013.04.003 PMC3677099 23643950

[63] Anderson P Kedersha N. RNA Granules: Post-transcriptional and epigenetic modulators of gene expression. Nat Rev Mol Cell Biol (2009) 10:430–6. doi: 10.1038/nrm2694 19461665

[64] Lloyd RE. How do viruses interact with stress-associated RNA granules? PloS Pathog (2012) 8:e1002741. doi: 10.1371/journal.ppat.1002741 22761570 PMC3386173

[65] Jefferson M Donaszi-Ivanov A Pollen S Dalmay T Saalbach G Powell PP. Host factors that interact with the pestivirus n-terminal protease, npro, are components of the ribonucleoprotein complex. J Virol (2014) 88:10340–53. doi: 10.1128/jvi.00984-14 PMC4178888 24965446

[66] Harman JL Loes AN Warren GD Heaphy MC Lampi KJ Harms MJ. Evolution of multifunctionality through a pleiotropic substitution in the innate immune protein S100A9. Elife (2020) 9:e54100. doi: 10.7554/eLife.54100 32255429 PMC7213983

[67] Vogl T Gharibyan AL Morozova-Roche LA. Pro-inflammatory S100A8 and S100A9 proteins: self-assembly into multifunctional native and amyloid complexes. Int J Mol Sci (2012) 13:2893–917. doi: 10.3390/ijms13032893 PMC3317694 22489132

[68] Källberg E Vogl T Liberg D Olsson A Björk P Wikström P. S100A9 interaction with TLR4 promotes tumor growth. PloS One (2012) 7:e34207. doi: 10.1371/journal.pone.0034207 22470535 PMC3314596

[69] Duan L Wu R Zhang X Wang D You Y Zhang Y. HBx-induced S100A9 in NF-κB dependent manner promotes growth and metastasis of hepatocellular carcinoma cells. Cell Death Dis (2018) 9:629. doi: 10.1038/s41419-018-0512-2 29795379 PMC5967311

[70] Schiopu A Cotoi OS. S100A8 and S100A9: DAMPs at the crossroads between innate immunity, traditional risk factors, and cardiovascular disease. Mediators Inflammation (2013) 2013:828354. doi: 10.1155/2013/828354 PMC3881579 24453429

[71] Laouedj M Tardif MR Gil L Raquil MA Lachhab A Pelletier M. S100A9 induces differentiation of acute myeloid leukemia cells through TLR4. Blood (2017) 129:1980–90. doi: 10.1182/blood-2016-09-738005 28137827

[72] He Z Riva M Björk P Swärd K Mörgelin M Leanderson T. CD14 is a Co-receptor for TLR4 in the S100A9-induced pro-inflammatory response in monocytes. PloS One (2016) 11:e0156377. doi: 10.1371/journal.pone.0156377 27228163 PMC4881898

[73] Lee NR Park BS Kim SY Gu A Kim DH Lee JS. Cytokine secreted by S100A9 *via* TLR4 in monocytes delays neutrophil apoptosis by inhibition of caspase 9/3 pathway. Cytokine (2016) 86:53–63. doi: 10.1016/j.cyto.2016.07.005 27459393

[74] Hadley RC Gu Y Nolan EM. Initial biochemical and functional evaluation of murine calprotectin reveals Ca(II)-dependence and its ability to chelate multiple nutrient transition metal ions. Biochemistry (2018) 57:2846–56. doi: 10.1021/acs.biochem.8b00309 PMC5953840 29659256

[75] Damo SM Kehl-Fie TE Sugitani N Holt ME Rathi S Murphy WJ. Molecular basis for manganese sequestration by calprotectin and roles in the innate immune response to invading bacterial pathogens. Proc Natl Acad Sci U.S.A. (2013) 110:3841–6. doi: 10.1073/pnas.1220341110 PMC3593839 23431180

[76] Clark HL Jhingran A Sun Y Vareechon C de Jesus Carrion S Skaar EP. Zinc and manganese chelation by neutrophil S100A8/A9 (Calprotectin) limits extracellular aspergillus fumigatus hyphal growth and corneal infection. J Immunol (2016) 196:336–44. doi: 10.4049/jimmunol.1502037 PMC4684987 26582948

[77] Nakashige TG Stephan JR Cunden LS Brophy MB Wommack AJ Keegan BC. The hexahistidine motif of host-defense protein human calprotectin contributes to zinc withholding and its functional versatility. J Am Chem Soc (2016) 138:12243–51. doi: 10.1021/jacs.6b06845 PMC5038136 27541598

[78] Nakashige TG Zhang B Krebs C Nolan EM. Human calprotectin is an iron-sequestering host-defense protein. Nat Chem Biol (2015) 11:765–71. doi: 10.1038/nchembio.1891 PMC4575267 26302479

[79] Hayden JA Brophy MB Cunden LS Nolan EM. High-affinity manganese coordination by human calprotectin is calcium-dependent and requires the histidine-rich site formed at the dimer interface. J Am Chem Soc (2013) 135:775–87. doi: 10.1021/ja3096416 PMC3575579 23276281

[80] Voss A Gescher K Hensel A Nacken W Zänker KS Kerkhoff C. Double-stranded RNA induces S100 gene expression by a cycloheximide-sensitive factor. FEBS Lett (2012) 586:196–203. doi: 10.1016/j.febslet.2011.12.022 22209981

[81] Tugizov S Berline J Herrera R Penaranda ME Nakagawa M Palefsky J. Inhibition of human papillomavirus type 16 E7 phosphorylation by the S100 MRP-8/14 protein complex. J Virol (2005) 79:1099–112. doi: 10.1128/jvi.79.2.1099-1112.2005 PMC538578 15613338

[82] Wang S Su M Lin J Zhang L Li J Tian Y. S100A8/A9, an upregulated host factor in BK virus infection after kidney transplantation, is associated with allograft function impairment. J Proteome Res (2022) 21:2356–66. doi: 10.1021/acs.jproteome.2c00219 36103633

[83] Undi RB Larabee JL Filiberti A Ulahannan S Aravindan S Stroberg E. Targeting doublecortin-like kinase 1 (DCLK1)-regulated SARS-CoV-2 pathogenesis in COVID-19. J Virol (2022) 96:e0096722. doi: 10.1128/jvi.00967-22 35943255 PMC9472619

[84] Darweesh MF Rajput MKS Braun LJ Rohila JS Chase CCL. BVDV npro protein mediates the BVDV induced immunosuppression through interaction with cellular S100A9 protein. Microb Pathog (2018) 121:341–9. doi: 10.1016/j.micpath.2018.05.047 PMC7127600 29859294

[85] Short KM Cox TC. Subclassification of the RBCC/TRIM superfamily reveals a novel motif necessary for microtubule binding. J Biol Chem (2006) 281:8970–80. doi: 10.1074/jbc.M512755200 16434393

[86] Munir M. TRIM proteins: Another class of viral victims. Sci Signal (2010) 3:jc2. doi: 10.1126/scisignal.3118jc2 20407122

[87] Ozato K Shin DM Chang TH Morse HC 3rd. TRIM family proteins and their emerging roles in innate immunity. Nat Rev Immunol (2008) 8:849–60. doi: 10.1038/nri2413 PMC3433745 18836477

[88] Meroni G Diez-Roux G. TRIM/RBCC, a novel class of 'single protein RING finger' E3 ubiquitin ligases. Bioessays (2005) 27:1147–57. doi: 10.1002/bies.20304 16237670

[89] Uchil PD Quinlan BD Chan WT Luna JM Mothes W. TRIM E3 ligases interfere with early and late stages of the retroviral life cycle. PloS Pathog (2008) 4:e16. doi: 10.1371/journal.ppat.0040016 18248090 PMC2222954

[90] Gack MU Shin YC Joo CH Urano T Liang C Sun L. TRIM25 RING-finger E3 ubiquitin ligase is essential for RIG-i-mediated antiviral activity. Nature (2007) 446:916–20. doi: 10.1038/nature05732 17392790

[91] Wang J Liu B Wang N Lee YM Liu C Li K. TRIM56 is a virus- and interferon-inducible E3 ubiquitin ligase that restricts pestivirus infection. J Virol (2011) 85:3733–45. doi: 10.1128/jvi.02546-10 PMC3126137 21289118

[92] Heidary F Gharebaghi R. Systematic review of the antiviral properties of TRIM56: A potential therapeutic intervention for COVID-19. Expert Rev Clin Immunol (2020) 16:973–84. doi: 10.1080/1744666X.2020.1822168 32903131

[93] Hiscott J Kwon H Génin P. Hostile takeovers: Viral appropriation of the NF-kappaB pathway. J Clin Invest (2001) 107:143–51. doi: 10.1172/jci11918 PMC199181 11160127

[94] Hiscott J Nguyen TL Arguello M Nakhaei P Paz S. Manipulation of the nuclear factor-kappaB pathway and the innate immune response by viruses. Oncogene (2006) 25:6844–67. doi: 10.1038/sj.onc.1209941 PMC7100320 17072332

[95] Santoro MG Rossi A Amici C. NF-kappaB and virus infection: Who controls whom. EMBO J (2003) 22:2552–60. doi: 10.1093/emboj/cdg267 PMC156764 12773372

[96] Huxford T Huang DB Malek S Ghosh G. The crystal structure of the IkappaBalpha/NF-kappaB complex reveals mechanisms of NF-kappaB inactivation. Cell (1998) 95:759–70. doi: 10.1016/s0092-8674(00)81699-2 9865694

[97] Jacobs MD Harrison SC. Structure of an IkappaBalpha/NF-kappaB complex. Cell (1998) 95:749–58. doi: 10.1016/s0092-8674(00)81698-0 9865693

[98] Pahl HL. Activators and target genes of Rel/NF-kappaB transcription factors. Oncogene (1999) 18:6853–66. doi: 10.1038/sj.onc.1203239 10602461

[99] Silverman N Maniatis T. NF-kappaB signaling pathways in mammalian and insect innate immunity. Genes Dev (2001) 15:2321–42. doi: 10.1101/gad.909001 11562344

[100] DiDonato JA Hayakawa M Rothwarf DM Zandi E Karin M. A cytokine-responsive IkappaB kinase that activates the transcription factor NF-kappaB. Nature (1997) 388:548–54. doi: 10.1038/41493 9252186

[101] Sakurai H Chiba H Miyoshi H Sugita T Toriumi W. IkappaB kinases phosphorylate NF-kappaB p65 subunit on serine 536 in the transactivation domain. J Biol Chem (1999) 274:30353–6. doi: 10.1074/jbc.274.43.30353 10521409

[102] Beg AA Ruben SM Scheinman RI Haskill S Rosen CA Baldwin AS Jr. I Kappa b interacts with the nuclear localization sequences of the subunits of NF-kappa b: a mechanism for cytoplasmic retention. Genes Dev (1992) 6:1899–913. doi: 10.1101/gad.6.10.1899 1340770

[103] Winston JT Strack P Beer-Romero P Chu CY Elledge SJ Harper JW. The SCFbeta-TRCP-ubiquitin ligase complex associates specifically with phosphorylated destruction motifs in IkappaBalpha and beta-catenin and stimulates IkappaBalpha ubiquitination *in vitro* . Genes Dev (1999) 13:270–83. doi: 10.1101/gad.13.3.270 PMC316433 9990852

[104] Zamanian-Daryoush M Mogensen TH DiDonato JA Williams BR. NF-kappaB activation by double-stranded-RNA-activated protein kinase (PKR) is mediated through NF-kappaB-inducing kinase and IkappaB kinase. Mol Cell Biol (2000) 20:1278–90. doi: 10.1128/mcb.20.4.1278-1290.2000 PMC85265 10648614

[105] Yoneyama M Fujita T. RNA Recognition and signal transduction by RIG-i-like receptors. Immunol Rev (2009) 227:54–65. doi: 10.1111/j.1600-065X.2008.00727.x 19120475

[106] Arenzana-Seisdedos F Turpin P Rodriguez M Thomas D Hay RT Virelizier JL. Nuclear localization of I kappa b alpha promotes active transport of NF-kappa b from the nucleus to the cytoplasm. J Cell Sci (1997) 110(Pt 3):369–78. doi: 10.1242/jcs.110.3.369 9057089

[107] Rodriguez MS Thompson J Hay RT Dargemont C. Nuclear retention of IkappaBalpha protects it from signal-induced degradation and inhibits nuclear factor kappaB transcriptional activation. J Biol Chem (1999) 274:9108–15. doi: 10.1074/jbc.274.13.9108 10085161

[108] Sachdev S Hoffmann A Hannink M. Nuclear localization of IkappaB alpha is mediated by the second ankyrin repeat: The IkappaB alpha ankyrin repeats define a novel class of cis-acting nuclear import sequences. Mol Cell Biol (1998) 18:2524–34. doi: 10.1128/mcb.18.5.2524 PMC110632 9566872

[109] Ting AT Bertrand MJM. More to life than NF-κB in TNFR1 signaling. Trends Immunol (2016) 37:535–45. doi: 10.1016/j.it.2016.06.002 PMC5076853 27424290

[110] Fiume G Vecchio E De Laurentiis A Trimboli F Palmieri C Pisano A. Human immunodeficiency virus-1 tat activates NF-κB *via* physical interaction with IκB-α and p65. Nucleic Acids Res (2012) 40:3548–62. doi: 10.1093/nar/gkr1224 PMC3333881 22187158

[111] Chen LJ Dong XY Zhao MQ Shen HY Wang JY Pei JJ. Classical swine fever virus failed to activate nuclear factor-kappa b signaling pathway both. Vitro vivo. Virol J (2012) 9:293. doi: 10.1186/1743-422x-9-293 23186553 PMC3565942

[112] Lees DM Hart IR Marshall JF. Existence of multiple isoforms of HS1-associated protein X-1 in murine and human tissues. J Mol Biol (2008) 379:645–55. doi: 10.1016/j.jmb.2008.04.020 18472110

[113] Carlsson G van't Hooft I Melin M Entesarian M Laurencikas E Nennesmo I. Central nervous system involvement in severe congenital neutropenia: Neurological and neuropsychological abnormalities associated with specific HAX1 mutations. J Intern Med (2008) 264:388–400. doi: 10.1111/j.1365-2796.2008.01982.x 18513342

[114] Suzuki Y Demoliere C Kitamura D Takeshita H Deuschle U Watanabe T. HAX-1, a novel intracellular protein, localized on mitochondria, directly associates with HS1, a substrate of src family tyrosine kinases. J Immunol (1997) 158:2736–44. 9058808

[115] Vafiadaki E Arvanitis DA Pagakis SN Papalouka V Sanoudou D Kontrogianni-Konstantopoulos A. The anti-apoptotic protein HAX-1 interacts with SERCA2 and regulates its protein levels to promote cell survival. Mol Biol Cell (2009) 20:306–18. doi: 10.1091/mbc.e08-06-0587 PMC2613088 18971376

[116] Johns HL Doceul V Everett H Crooke H Charleston B Seago J. The classical swine fever virus n-terminal protease n(pro) binds to cellular HAX-1. J Gen Virol (2010) 91:2677–86. doi: 10.1099/vir.0.022897-0 20631090

[117] Vafiadaki E Sanoudou D Arvanitis DA Catino DH Kranias EG Kontrogianni-Konstantopoulos A. Phospholamban interacts with HAX-1, a mitochondrial protein with anti-apoptotic function. J Mol Biol (2007) 367:65–79. doi: 10.1016/j.jmb.2006.10.057 17241641

[118] Chkheidze AN Lyakhov DL Makeyev AV Morales J Kong J Liebhaber SA. Assembly of the alpha-globin mRNA stability complex reflects binary interaction between the pyrimidine-rich 3' untranslated region determinant and poly(C) binding protein alphaCP. Mol Cell Biol (1999) 19:4572–81. doi: 10.1128/mcb.19.7.4572 PMC84255 10373506

[119] Holcik M Liebhaber SA. Four highly stable eukaryotic mRNAs assemble 3' untranslated region RNA-protein complexes sharing cis and trans components. Proc Natl Acad Sci U.S.A. (1997) 94:2410–4. doi: 10.1073/pnas.94.6.2410 PMC20101 9122208

[120] Wang X Kiledjian M Weiss IM Liebhaber SA. Detection and characterization of a 3' untranslated region ribonucleoprotein complex associated with human alpha-globin mRNA stability. Mol Cell Biol (1995) 15:1769–77. doi: 10.1128/mcb.15.3.1769 PMC230401 7862166

[121] Kim SS Pandey KK Choi HS Kim SY Law PY Wei LN. Poly(C) binding protein family is a transcription factor in mu-opioid receptor gene expression. Mol Pharmacol (2005) 68:729–36. doi: 10.1124/mol.105.012245 15933215

[122] Ko JL Loh HH. Poly c binding protein, a single-stranded DNA binding protein, regulates mouse mu-opioid receptor gene expression. J Neurochem (2005) 93:749–61. doi: 10.1111/j.1471-4159.2005.03089.x 15836633

[123] Meng Q Rayala SK Gururaj AE Talukder AH O'Malley BW Kumar R. Signaling-dependent and coordinated regulation of transcription, splicing, and translation resides in a single coregulator, PCBP1. Proc Natl Acad Sci U.S.A. (2007) 104:5866–71. doi: 10.1073/pnas.0701065104 PMC1851583 17389360

[124] Blyn LB Towner JS Semler BL Ehrenfeld E. Requirement of poly(rC) binding protein 2 for translation of poliovirus RNA. J Virol (1997) 71:6243–6. doi: 10.1128/jvi.71.8.6243-6246.1997 PMC191892 9223526

[125] Gamarnik AV Andino R. Two functional complexes formed by KH domain containing proteins with the 5' noncoding region of poliovirus RNA. Rna (1997) 3:882–92. PMC1369533 9257647

[126] Makeyev AV Liebhaber SA. The poly(C)-binding proteins: A multiplicity of functions and a search for mechanisms. Rna (2002) 8:265–78. doi: 10.1017/s1355838202024627 PMC1370249 12003487

[127] Huang C Jiang T Xue M Li Y Feng T Pan W. Poly(C)-binding protein 2 positively regulates interferon downstream signaling. Acta Biochim Biophys Sin (Shanghai) (2022) 54:748–51. doi: 10.3724/abbs.2022032 PMC9828312 35593468

[128] Michael WM Eder PS Dreyfuss G. The K nuclear shuttling domain: A novel signal for nuclear import and nuclear export in the hnRNP K protein. EMBO J (1997) 16:3587–98. doi: 10.1093/emboj/16.12.3587 PMC1169983 9218800

[129] Li D Dong H Li S Munir M Chen J Luo Y. Hemoglobin subunit beta interacts with the capsid protein and antagonizes the growth of classical swine fever virus. J Virol (2013) 87:5707–17. doi: 10.1128/jvi.03130-12 PMC3648164 23487454

[130] Mordstein M Neugebauer E Ditt V Jessen B Rieger T Falcone V. Lambda interferon renders epithelial cells of the respiratory and gastrointestinal tracts resistant to viral infections. J Virol (2010) 84:5670–7. doi: 10.1128/jvi.00272-10 PMC2876583 20335250

[131] Onoguchi K Yoneyama M Takemura A Akira S Taniguchi T Namiki H. Viral infections activate types I and III interferon genes through a common mechanism. J Biol Chem (2007) 282:7576–81. doi: 10.1074/jbc.M608618200 17204473

[132] Thomson SJ Goh FG Banks H Krausgruber T Kotenko SV Foxwell BM. The role of transposable elements in the regulation of IFN-lambda1 gene expression. Proc Natl Acad Sci U.S.A. (2009) 106:11564–9. doi: 10.1073/pnas.0904477106 PMC2710658 19570999

[133] Sommereyns C Paul S Staeheli P Michiels T. IFN-lambda (IFN-lambda) is expressed in a tissue-dependent fashion and primarily acts on epithelial cells *in vivo* . PloS Pathog (2008) 4:e1000017. doi: 10.1371/journal.ppat.1000017 18369468 PMC2265414

[134] Cao T Li X Xu Y Zhang S Wang Z Shan Y. Npro of classical swine fever virus suppresses type III interferon production by inhibiting IRF1 expression and its nuclear translocation. Viruses (2019) 11(11):998. doi: 10.3390/v11110998 31683525 PMC6893713

[135] Odendall C Dixit E Stavru F Bierne H Franz KM Durbin AF. Diverse intracellular pathogens activate type III interferon expression from peroxisomes. Nat Immunol (2014) 15:717–26. doi: 10.1038/ni.2915 PMC4106986 24952503

[136] Mätzener P Magkouras I Rümenapf T Peterhans E Schweizer M. The viral RNase e(rns) prevents IFN type-I triggering by pestiviral single- and double-stranded RNAs. Virus Res (2009) 140:15–23. doi: 10.1016/j.virusres.2008.10.015 19041350

[137] Lussi C Schweizer M. What can pestiviral endonucleases teach us about innate immunotolerance? Cytokine Growth Factor Rev (2016) 29:53–62. doi: 10.1016/j.cytogfr.2016.03.003 27021825 PMC7173139

[138] Tews BA Klingebeil A Kühn J Franzke K Rümenapf T Meyers G. The e(rns) carboxyterminus: Much more than a membrane anchor. Viruses (2021) 13(7):1203. doi: 10.3390/v13071203 34201636 PMC8310223

